# Factors Determining Black Carbon Exposures among Pregnant Women Enrolled in the HAPIN Trial

**DOI:** 10.1021/acs.est.3c09991

**Published:** 2024-05-29

**Authors:** Devan A. Campbell, Michael Johnson, Ricardo Piedrahita, Ajay Pillarisetti, Lance A. Waller, Katherine A. Kearns, Jacob Kremer, Erick Mollinedo, Jeremy A. Sarnat, Maggie L. Clark, Lindsay J. Underhill, John P. McCracken, Anaité Diaz-Artiga, Kyle Steenland, Ghislaine Rosa, Miles A. Kirby, Kalpana Balakrishnan, Sankar Sambandam, Krishnendu Mukhopadhyay, Saritha Sendhil, Amudha Natarajan, Florien Ndagijimana, Ephrem Dusabimana, Lisa M. Thompson, William Checkley, Laura Nicolaou, Stella Hartinger, Jennifer L. Peel, Thomas F. Clasen, Luke P. Naeher

**Affiliations:** † University of Georgia, Athens, Georgia 30602, United States; ‡ Berkeley Air Monitoring Group, Berkeley, California 94701, United States; § Environmental Health Sciences, School of Public Health, University of California, Berkeley, California 94720, United States; ∥ Department of Biostatistics and Bioinformatics, Rollins School of Public Health, Emory University, Atlanta, Georgia 80521, United States; ⊥ Department of Environmental and Radiological Health Sciences, Colorado State University, Fort Collins, Colorado 80523, United States; # Washington University School of Medicine, St. Louis, Missouri 63110, United States; ∇ Center for Health Studies, Universidad del Valle de Guatemala, Guatemala City, Guatemala 01015, United States; ○ Gangarosa Department of Environmental Health, Rollins School of Public Health, Emory University, Atlanta, Georgia 30322, United States; ◆ Department of Public Health, Policy and Systems, University of Liverpool, Liverpool L69 3GF, U.K.; ¶ Department of Global Health and Population, Harvard T.H. Chan School of Public Health, Boston, Massachusetts 02115, United States; †† ICMR Center for Advanced Research on Air quality, Climate and Health, Department of Environmental Health Engineering, Sri Ramachandra Institute of Higher Education and Research, Chennai 600001, India; ‡‡ Eagle Research Center, Kigali, Rwanda 00000, United States; §§ Nell Hodgson Woodruff School of Nursing, Emory University, Atlanta, Georgia 30322, United States; ∥∥ Center for Global Non-Communicable Diseases, Johns Hopkins University, Baltimore, Maryland 21205, United States; ⊥⊥ Division of Pulmonary and Critical Care, School of Medicine, Johns Hopkins University, Baltimore, Maryland 21205, United States; ## Benchmark Risk Group, Chicago, Illinois 60601, United States

**Keywords:** household air pollution, exposure assessment, exposure models, intervention, black carbon (BC), biomass fuel stoves, liquefied petroleum gas, Guatemala, India, Peru, Rwanda

## Abstract

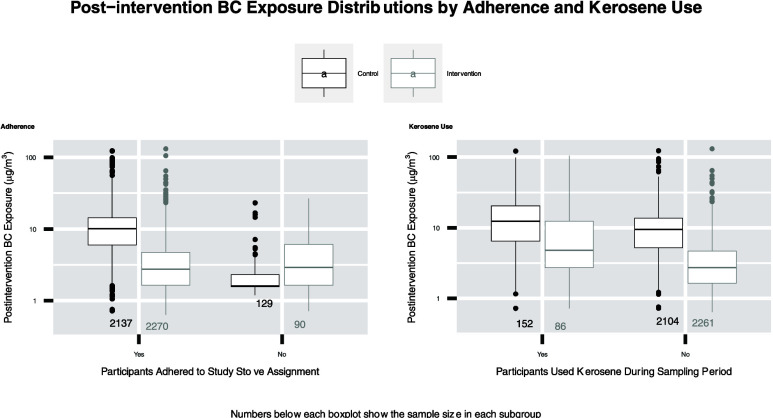

Residential biomass burning is an important source of black carbon (BC) exposure among rural communities in low- and middle-income countries. We collected 7165 personal BC samples and individual/household level information from 3103 pregnant women enrolled in the Household Air Pollution Intervention Network trial. Women in the intervention arm received free liquefied petroleum gas stoves and fuel throughout pregnancy; women in the control arm continued the use of biomass stoves. Median (IQR) postintervention BC exposures were 9.6 μg/m^3^ (5.2–14.0) for controls and 2.8 μg/m^3^ (1.6–4.8) for the intervention group. Using mixed models, we characterized predictors of BC exposure and assessed how exposure contrasts differed between arms by select predictors. Primary stove type was the strongest predictor (*R*^2^ = 0.42); the models including kerosene use, kitchen location, education, occupation, or stove use hours also provided additional explanatory power from the base model adjusted only for the study site. Our full, trial-wide, model explained 48% of the variation in BC exposures. We found evidence that the BC exposure contrast between arms differed by study site, adherence to the assigned study stove, and whether the participant cooked. Our findings highlight factors that may be addressed before and during studies to implement more impactful cookstove intervention trials.

## Abstract 2

Our study adds to the scientific discourse on understanding and mitigating black carbon exposures in resource-constrained settings.

## Introduction

Approximately 2.3 billion people are exposed to household air pollution due to the residential combustion of polluting biomass fuels (e.g., wood, dung, charcoal), coal, and kerosene used for household energy needs.^1^ Exposure to household air pollution ranks among the top risk factors contributing to the global burden of disease and accounted for 2.3 million deaths and more than 90 million disability-adjusted life years (DALYs) in 2019 with disproportionate adverse health effects in low- and middle-income countries (LMICs).^2^ Health outcomes associated with household air pollution include adverse respiratory (acute respiratory infection in both adults and children, chronic obstructive pulmonary disease, and lung cancer), cardiovascular (cerebrovascular disease, ischemic heart disease, and cardiovascular mortality), and birth (low birth weight and small for gestational age) outcomes.^3−11^

Household air pollution is primarily characterized via measurements of particulate matter with an aerodynamic diameter of ≤2.5 μm (PM_2.5_) and carbon monoxide (CO).^12^ PM_2.5_ is a heterogeneous chemical mixture containing elemental carbon (EC), commonly referred to as black carbon (BC), organic carbon (OC), polycyclic aromatic hydrocarbons (PAHs), and various metal species.^13^ BC, the most light-absorbing portion of PM_2.5_, is a byproduct of incomplete combustion and is therefore a more specific marker of combustion-related sources than PM_2.5_ alone. BC has been gaining attention due to its global warming potential^14^ and its associations with adverse health effects.^15,16^ Residential biomass burning is the top-ranked source of BC globally (∼35%) with increasing emissions^17^ due to growing rural populations in LMICs.^18^ More studies of BC in LMICs are needed to better gauge its contribution to adverse climate impacts and human health.

To date, BC remains largely understudied in settings where polluting fuels are used for daily cooking. The majority of epidemiology studies linking BC to health outcomes have been conducted in Europe and North America.^19,20^ Many health and exposure studies in LMICs have reported the effectiveness of cookstove interventions in providing meaningful health benefits or reductions in exposure to household air pollution.^21−27^ Few cookstove intervention trials have assessed personal BC exposures.^23,28,29^

While several space- and time-dependent factors potentially impact PM_2.5_ exposures due to household air pollution (e.g., stove type, kitchen location, time spent cooking), little is known about how these factors are associated with personal BC exposures.^30^ Findings from a global exposure assessment of 2541 households across 8 LMICs from the PURE-AIR cohort suggest that BC concentrations vary according to study site and primary and secondary cooking fuels used.^31^ A more comprehensive assessment of 870 individuals from the PURE-AIR cohort found that stove and fuel type, hours spent cooking, and having a window in the kitchen were important factors influencing personal BC exposure.^32^ The study did not collect longitudinal data nor did it address potentially substantial contributions from kerosene used for lighting.^32^ Other studies of BC exposures among individuals in LMICs identified roof type, number of bedrooms, ventilation, heating and lighting fuel, kitchen type, temperature, and ambient air pollutant concentrations to be significant predictors of personal BC, though these studies are cross-sectional and/or limited in their geographical scope.^33−35^ There is a need for longitudinal analyses across multiple settings to fully elucidate how space- and time-dependent factors impact BC exposures in resource-limited settings where exposure to household air pollution is substantial.

The Household Air Pollution Intervention Network (HAPIN) randomized controlled trial provided liquefied petroleum gas (LPG) stoves and continuous free fuel distribution in four LMICs.^36^ Details on personal exposures to PM_2.5_, BC, and CO among pregnant women in the HAPIN trial, both overall and by intervention arm and study site, have been published previously.^37^ Here, we explore the association between BC exposure and a variety of household characteristics, participant practices, and other factors assessed at baseline and follow-up visits. To complement this analysis, we also evaluate how select factors potentially modified the effectiveness of the LPG intervention in reducing personal BC exposures in the HAPIN trial.

## Methods

### Study Setting

The HAPIN study (registration NCT02944682) was a randomized controlled trial evaluating the health effects of an introduced LPG stove and free fuel intervention versus the continued use of traditional cookstoves in Guatemala, India, Peru, and Rwanda.^36^ In each study site, ∼800 households were enrolled totaling 3195 pregnant women (aged 18–35 years) randomized to either control or intervention arm.^36^ As part of HAPIN, we analyzed 24 h personal BC exposures from the 3103 (97%) pregnant women with at least one valid measurement (*n* = 7,165 BC measures) across the four study sites.

HAPIN specifically chose rural communities without other major ambient air pollution sources and a high proportion of homes that typically use traditional biomass cookstoves for cooking and heating purposes to reflect locations where a household air pollution intervention would be expected to provide maximum exposure reductions. Details regarding study site characteristics, inclusion and exclusion criteria, and overall study design are described elsewhere.^36,38^ Pregnancy-related exposures as well as details on total versus valid samples collected during pregnancy are reported in our previous work.^37^ We provide a brief description of the HAPIN exposure sampling plan during pregnancy below.

### Exposure Measurement

24 h average exposure measurements were conducted three times during pregnancy. Baseline measures were collected at 9–20 weeks of gestation before the LPG stove was introduced. Two follow-up measurements were made at 24–28 (postintervention visit 1) and 32–36 (postintervention visit 2) weeks of gestation. At each visit, participants were asked to wear customized vests or aprons fitted with an air-monitoring instrument near their breathing zone. If participants were to conduct activities that could damage the equipment, they were asked to remove the vest but keep it nearby (within 1–2m). Wearing compliance for our study participants was reported in our previous work.^37^

PM_2.5_ was collected using the RTI Enhanced Children’s MicroPEM (ECM, RTI International, Research Triangle Park). The ECM uses a 2.5 μm size-cut impactor at a flow rate of 0.3 L/min to collect gravimetric samples on 15 mm poly(tetrafluoroethylene) (Teflon) filters (Measurement Technology Laboratories). The ECM also logs temperature, relative humidity, and triaxial accelerometry.

BC concentrations from PM_2.5_ filter samples were estimated using the Sootscan Model OT21 Optical Transmissometers (Magee Scientific) at either the University of Georgia (UGA, Athens, GA), for samples collected in Guatemala, Peru, and Rwanda, or at Sri Ramachandra Institute for Higher Education and Research (SRIHER, Chennai, India), for samples collected in India. We measured the optical transmittance of filters and compared the intensity of light through a postsampled and reference filter. Over 97% of the reference filters, at UGA, were the presampled filters themselves, while at SRIHER, the reference filter was a blank laboratory filter.^37^

To estimate BC concentrations, we used an equation derived from Presler-Jur et al., 2017 that measures particle light absorption (eq 1):
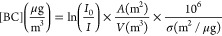
1Here, [BC] is the BC mass concentration, *I*_0_ is the intensity of light through a reference filter, *I* is the intensity of light through a postsampled filter, *A* is the area of the filter, *V* is the air sample volume, and σ is the attenuation cross section. This equation relates absorbance and mass content. We used the attenuation cross section value reported in Garland et al., 2017 (σ = 13.7 μg/cm^2^) for Teflon filters collected from similar source types.

### Data Collection on Individual and Household Characteristics

At the baseline visit, trained local field technicians administered questionnaires to assess baseline participant characteristics (e.g., age, family size, and access to electricity) and sources of household exposures (e.g., primary stove and fuel types used for cooking, heating, and lighting). At all exposure visits, including at baseline, technicians also collected information regarding behavioral practices that occurred during the prior 24 h sampling period (e.g., whether the participant cooked or not, stove and fuels used, any use of kerosene, self-reported hours of primary stove use, etc.) as well as additional household characteristics such as food insecurity, kitchen location, roof type, and the self-reported smoke from a source other than the participant’s stove. Primary stove types were categorized as open fires or LPG stoves, chimney stoves, or other improved biomass stoves such as portable biomass stoves, comals (i.e., smooth, flat griddles typically used in Central America), charcoal-burning Imbabura stoves, and wood-burning Rondereza stoves. Our questionnaires were not specific enough for us to discern whether kerosene fuel was used explicitly for lighting or cooking activities during the sampling period. Seasonality was dichotomized into dry and rainy seasons according to the respective climate and weather patterns in Guatemala,^40^ India,^41^ Peru,^42^ and Rwanda.^43^ More in-depth descriptions of individual and household characteristics are provided in Text S1.

### Statistical Analysis

We conducted a descriptive analysis of the study population at baseline. Summary statistics for BC exposures according to select combustion-related factors expected and postintervention BC exposures among the control and intervention groups are provided in the Supporting Information. For all further analyses via linear models, we natural log-transformed BC exposures to meet regression assumptions. We conducted all analyses in *R* (versions 3.6 and 4.0, *R* Foundation for Statistical Computing, Vienna, Austria).

To assess the association between covariates and personal BC, we employed a series of linear mixed-effects models with random intercepts for participants to account for the correlation of repeated measurements made on the same participant. We ran mixed models for all HAPIN measures (adjusting for study site) and for each study site separately to look at univariate associations between covariates and exposure; results are provided in Supporting Information (eq 2). Here, *y*_*ij*_ is the *j*th measurement of BC for participant *i*; β_0_ is the overall intercept (mean); β_1_ is the coefficient for the study site adjustment (not included in the site-specific models); β_2_ is the coefficient for the covariate (*X*_2_) of interest in the model; α_*i*_ is the random intercept for participant *i*; and ε_*ij*_ is the random error. These models assume that α_*i*_ and ε_*ij*_ are independent and normally distributed with variances σ_b_^2^ (i.e., variance between individuals) and σ_w_^2^ (i.e., variance within individuals), respectively. HAPIN-wide bivariate models where the marginal *R*^2^ values are greater than that from the HAPIN-wide univariate study site model show instances where a covariable provided additional explanatory power. We then ran multivariable mixed-effects regression analyses to assess this relationship after adjusting for other model covariates (eq 3). Covariates were selected to be included in the model using a backward stepwise regression procedure that eliminated nonsignificant (*p* > 0.05) variables. A brief description of our analysis with imputed missing questionnaire data is provided in Text S2.
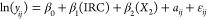
2
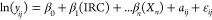
3All models were constructed, selected, validated, and analyzed using the lme4^44^ and sjPlot^45^ packages in *R*. Additionally, for each model, we estimated the root-mean-square error (RMSE) with the rmse^46^ package in *R* and obtained the marginal *R*^2^ value and intraclass correlation (ICC) using the sjPlot^45^ package in *R*. We calculated the RMSE as a metric for the absolute fit of the model to the data by taking the square root of the average of the squared residuals. The marginal *R*^2^ value represents the proportion of the variance in personal BC explained only by the fixed effects of the predictors included in the model. We determined the ICC, conditional on the predictors in the model, by estimating the proportion of the total variance in personal BC attributable to the variance between participants (eq 4).
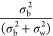
4We constructed linear mixed-effects models with an interaction term between the arm and all covariates included in the full models to assess which predictors potentially modified the effectiveness of the intervention (eq 5). Parameters with significant interaction coefficients were deemed to show potential effect modification. Next, we evaluated each predictor with significant interaction coefficients, separately, after controlling for all other covariates found to be associated with personal BC in previous models (eq 6). Although not completely comparable to BC exposure contrasts between arms reported in Johnson et al.,^37^ which only controlled for study site in the HAPIN-wide model, deviations from Johnson et al.^37^ results show how select predictors potentially modified the effect of the intervention in reducing exposures to BC. We used the emmeans package in *R* to compute and compare the marginal means in each sublevel of the treatment arm.^47^ For this analysis, we categorized self-reported stove use hours and relative humidity by quantile (i.e., ≤25th percentile, middle 50%, ≥75th percentile) and replaced primary stove type with a dichotomous indicator for adherence to the stove assignment in each treatment arm. Participants in the control and intervention arms adhered to the stove assignment if they, respectively, used a biomass or LPG stove as their primary stove.
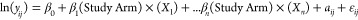
5
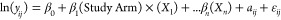
6

## Results

### Summary of Study Population

Baseline participant and household characteristics are provided in Table 1. The majority (70%) of our participants ranged in age from 20 to 29 years. Wood was the predominant cooking fuel used at baseline in Guatemala (99%) and India (100%). Cow dung was the most common cooking fuel used in Peru (87%); in Rwanda, participants used both wood (73%) and charcoal (25%). Electricity was the predominant primary lighting source in all study sites except for Rwanda where only 28% of the participants used electricity as their primary lighting source. Summary statistics for personal BC measurements overall and by select combustion-related factors are provided in Table S1. The majority of our 7165 personal BC measures were from participants using either open fires (49%) or LPG stoves (34%) as their primary stove type. Overall, the median (IQR) BC exposure level was 7.1 μg/m^3^ (2.9–12.6), and exposures ranged from 0.6 to 132.6 μg/m^3^. After restricting the data to postintervention study visits, median (IQR) BC exposures were 9.6 μg/m^3^ (5.2–14.0) and 2.8 μg/m^3^ (1.6–4.8) in the control and intervention arms, respectively (Table S2). Our complete data set, comprising both questionnaire/field observation and exposure data, includes approximately 81% (*n* = 5791) of our total BC measures. We have missing data for kitchen volume (13%), roof material (12%), wall material (12%), temperature and relative humidity (5%), hours of self-reported stove use during the 24 h sampling period (2%), food security (2%), kitchen location (2%), participant occupation (<1%), other sources of smoke (1%), lighting fuel (<1%), fuel type (<1%), and kerosene use (<1%) (Figure S1).

**Table 1:** Summary of Baseline Participant and Household Characteristics of the 3103 Women Included in the Study

		HAPIN	Guatemala	India	Peru	Rwanda
characteristic	category	N (%)	N (%)	N (%)	N (%)	N (%)
total		3103 (100%)	791 (100%)	787 (100%)	753 (100%)	772 (100%)
age	<20	388 (13%)	119 (15%)	126 (16%)	94 (12%)	49 (6%)
	20–24	1161 (37%)	320 (40%)	380 (48%)	264 (35%)	197 (26%)
	25–29	979 (32%)	226 (29%)	223 (28%)	245 (33%)	285 (37%)
	30–35	568 (18%)	122 (15%)	58 (7%)	149 (20%)	239 (31%)
primary cooking fuel	charcoal	190 (6%)				190 (25%)
	cow dung	656 (21%)			656 (87%)	
	other	30 (1%)	3 (0%)		9 (1%)	18 (2%)
	wood	2220 (72%)	784 (99%)	787 (100%)	87 (12%)	562 (73%)
	missing	7 (0%)	4 (1%)		1 (0%)	2 (0%)
primary lighting source	electricity	2362 (76%)	697 (88%)	757 (96%)	688 (91%)	220 (28%)
	kerosene lamp	81 (3%)	6 (1%)	16 (2%)	1 (0%)	58 (8%)
	other	179 (6%)	72 (9%)		30 (4%)	77 (10%)
	solar light	275 (9%)	2 (0%)	3 (0%)	22 (3%)	248 (32%)
	torch (battery)	199 (6%)	10 (1%)	11 (1%)	11 (1%)	167 (22%)
participant’s occupation	agriculture	1479 (48%)	6 (1%)	333 (42%)	568 (75%)	572 (74%)
	commercial	126 (4%)	17 (2%)	4 (1%)	23 (3%)	82 (11%)
	household	1335 (43%)	733 (93%)	425 (54%)	121 (16%)	56 (7%)
	other	154 (5%)	29 (4%)	25 (3%)	40 (5%)	60 (8%)
	missing	9 (0%)	6 (1%)			
family size	small (≤4)	1994 (64%)	386 (49%)	572 (73%)	386 (49%)	617 (80%)
	medium (5–9)	1022 (33%)	340 (43%)	212 (27%)	340 (43%)	148 (19%)
	large (>10)	79 (3%)	61 (8%)	3 (0%)	61 (8%)	5 (1%)
	missing	8 (0%)			4 (1%)	
household food insecurity	none	1743 (56%)	435 (55%)	634 (81%)	388 (52%)	286 (37%)
	mild	835 (27%)	249 (31%)	113 (14%)	259 (34%)	214 (28%)
	moderate/severe	474 (15%)	93 (12%)	36 (5%)	95 (13%)	250 (32%)
	missing	51 (2%)	14 (2%)	4 (1%)	11 (1%)	22 (3%)
participant’s education	no complete formal education	804 (32%)	311 (46%)	235 (34%)	24 (4%)	234 (41%)
	primary school complete	861 (34%)	268 (40%)	195 (28%)	168 (28%)	230 (40%)
	secondary school or equivalent complete	873 (34%)	95 (14%)	269 (38%)	404 (68%)	105 (18%)
	missing	1 (0%)	1 (0%)	0 (0%)	0 (0%)	0 (0%)

### Major Predictors of Black Carbon Exposures

HAPIN-wide (adjusted for study site) and site-specific associations between personal BC and single household, individual, and environmental characteristics, unadjusted for other covariates, are provided in Table S3. Primary stove type and study site explained the most variation in personal BC among all other covariates in our HAPIN-wide analysis (*R*^2^ = 0.42), and stove type remained important in each site-specific model (*R*^2^ range 0.36–0.48). The HAPIN-wide models including kerosene use, kitchen location, participant occupation, participant education, and stove use hours provided additional explanatory power compared to the model including only the study site with *R*^2^ values ranging from 0.09 to 0.1. Fuel type had considerable explanatory power at baseline in Rwanda (*R*^2^ = 0.21) where charcoal users had lower (−48, 95% CI: −52, −41) BC exposures than wood users. However, in Peru, there was no significant difference in BC exposures among wood and cow dung users at baseline.

**Figure 1:**
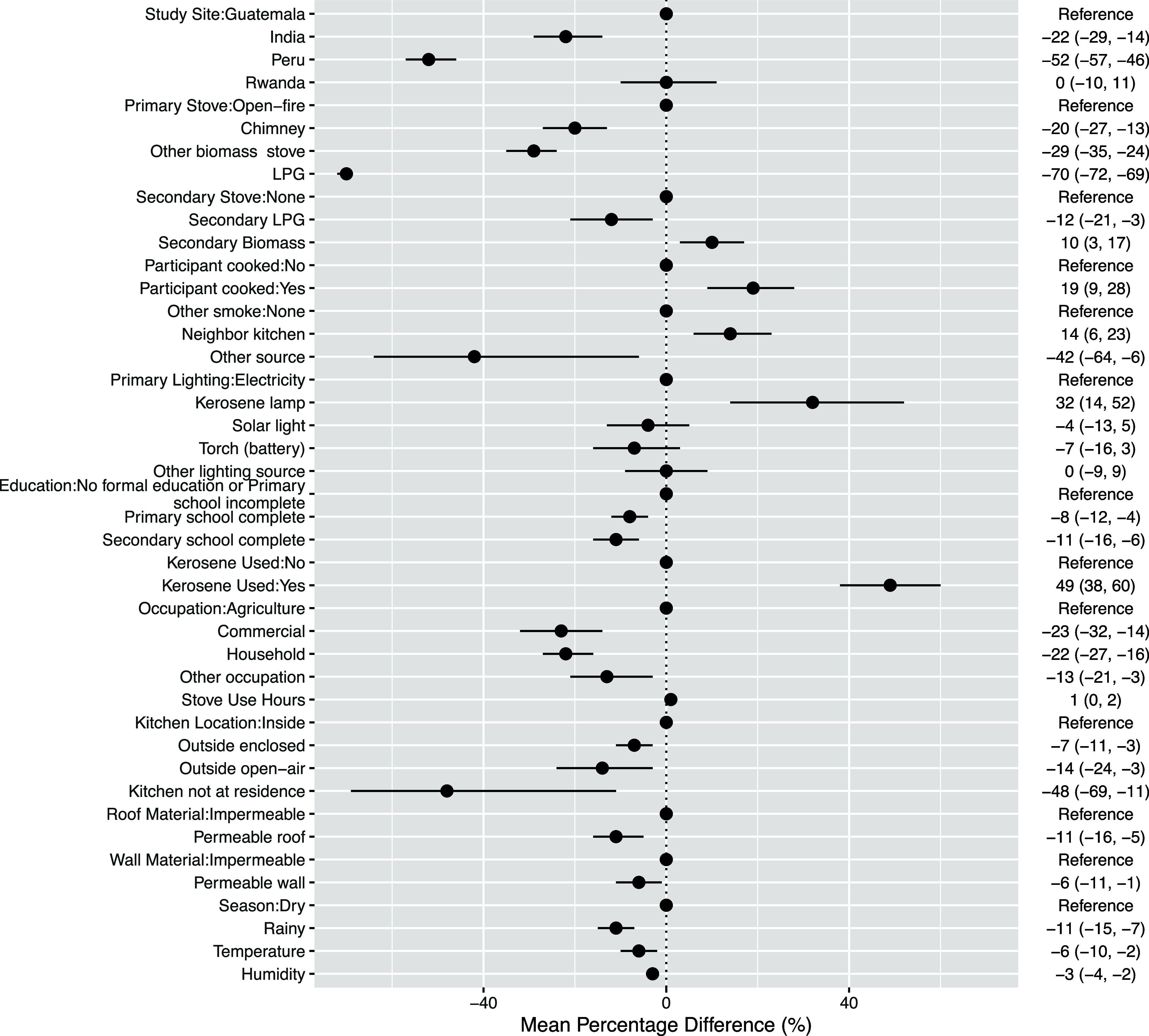
HAPIN-wide multivariable mixed-effects regression coefficients (with 95% confidence intervals). Numeric coefficients represent the mean percentage change of the geometric mean in BC exposure compared to the reference category based on the final multivariable linear regression models. Coefficients for relative humidity and kitchen volume represent a 5 unit increase in percentage and 10 unit increase in volume, respectively.

HAPIN-wide multivariable mixed regression coefficients between personal BC and select factors are provided in Figure 1; site-specific coefficients are provided in Figures S2–S5. We observed variation between study sites as BC exposures were lower in India (−22, 95% CI: −29, −14) and Peru (−52, 95% CI: −57, −46), compared to Guatemala. However, the most variation in BC exposures was observed as an exposure gradient according to the primary stove and fuel types: compared to traditional open fires, BC was lower for LPG (−70, 95% CI: −72, −69), followed by other improved biomass stoves (−29, 95% CI: −35, −24), and chimney stoves (−20, 95% CI: −27, −13), respectively. Additionally, participants with secondary LPG (−12, 95% CI: −21, −3) or biomass (10, 95% CI: 3, 17) stoves had lower and higher BC exposures than those with no secondary stoves, respectively. General use of kerosene was associated with an increase (49, 95% CI: 38, 60) in personal BC across HAPIN and was included in each site-specific model except Peru, which had minimal kerosene users. In these models, any kerosene use, versus no use of kerosene, was associated with an increase in personal BC in Guatemala (22, 95% CI: 1, 48), India (50, 95% CI: 36, 65), and Rwanda (63, 95% CI: 39, 92). Kerosene lamp users had 32% (95% CI: 14, 52) higher BC exposures than those that used electricity for lighting. Another combustion-related variable impacting BC exposures was self-reported smoke from a neighbor’s kitchen, which was associated with elevated BC exposures (14, 95% CI: 6, 23) compared to when no other sources of outside smoke were reported.

We also observed associations between personal BC and other individual and household characteristics as well as environmental conditions. We observed an exposure gradient according to kitchen location: compared to participants with kitchens located inside the home, BC exposures were lower for those with outside enclosed kitchens (−7, 95% CI: −11, −3), outside open-air kitchens (−14, 95% CI: −24, −3), and kitchens that were not located at the residence (−48, 95% CI: −69, −11) respectively. Participants who worked either in the household (−22, 95% CI: −27, −16) or as vendors (−23, 95% CI: −32, −14) had lower BC exposures compared to those working in agriculture. Participants with permeable roof (−11%, 95% CI: −16, −5) and wall (−6, 95% CI: −11, −1) materials had lower BC exposures than those with impermeable roof and wall materials. Each additional hour of stove use was positively associated with personal BC (1, 95% CI: 0, 2). BC exposures were found to be lower for participants with primary (−8, 95% CI: −12, −4) or secondary (−11, 95% CI: −16, −6) school education, compared to those with no formal education. Every 5% increase in relative humidity was negatively associated with personal BC (−3, 95% CI: −4, −2). We also observed every 5 °C increase in temperature to be negatively associated with personal BC (−6, 95% CI: −10, −2) and found BC exposures to be lower in the rainy season (−11, 95% CI: −15, −7) compared to the dry season. The rainy season was also associated with lower BC exposures, compared to the dry season, in Guatemala (−6, 95% CI: −10, 0) and Rwanda (−23, 95% CI: −30, −17).

### Model Performance and Fit

Model performance and fit statistics are provided in Table 2 and are comparable to those from models with imputed data for missing covariates (Table S4). Our HAPIN-wide model explained 48% of the variation in BC exposures among our study population with similar marginal *R*^2^ values for each site-specific model. The within-individual variance in personal BC was much greater than the between-individual variance as our ICC was 0.20 in the full HAPIN model. The RMSE between the natural logged-transformed predicted and measured BC exposures was 7.7 μg/m^3^.

**Table 2:** Measured and Modeled Exposure Summaries with Linear Mixed Model Statistics^a^

		measured exposure				modeled exposure				model statistics		
study site	model parameters	sample size	median (μg/m^3^)	mean (μg/m^3^)	SD (μg/m^3^)	sample size	median (μg/m^3^)	mean (μg/m^3^)	SD (μg/m^3^)	RMSE (μg/m^3^)	ICC	marginal *R*^2^
HAPIN	study site + primary stove type + secondary stove type + participant cooked + other sources of smoke + primary lighting source + education + general kerosene use + occupation + stove use hours + kitchen location + roof material + wall material + season + temperature + humidity	7165	7.1	9.3	9.5	5791	7.2	6.2	2.0	7.7	0.20	0.48
Guatemala	primary stove type + secondary stove type + other sources of smoke + general kerosene use + kitchen volume + humidity + season	2000	9.2	10	8.4	1799	9.7	7.8	1.7	6.9	0.25	0.50
India	primary stove type + primary lighting source + general kerosene use + occupation + wall material + stove use hours + season + temperature + humidity	1874	6.3	9.6	10.7	1784	6.6	5.8	2.1	8.7	0.18	0.45
Peru	primary stove type + secondary stove type + stove use hours + participant cooked + kitchen location + age at baseline + family size	1553	2.4	7.5	10.3	1516	4.5	4.0	2.1	8.3	0.24	0.46
Rwanda	primary stove type + participant cooked + other sources of smoke + general kerosene use + occupation + food insecurity + kitchen volume + season	1738	7.9	9.7	8.4	1142	8.1	7.4	1.6	5.8	0.06	0.49

a SD = standard deviation; RMSE = root mean square error; ICC = intraclass correlation.

### Potential for Effect Modification

**Figure 2:**
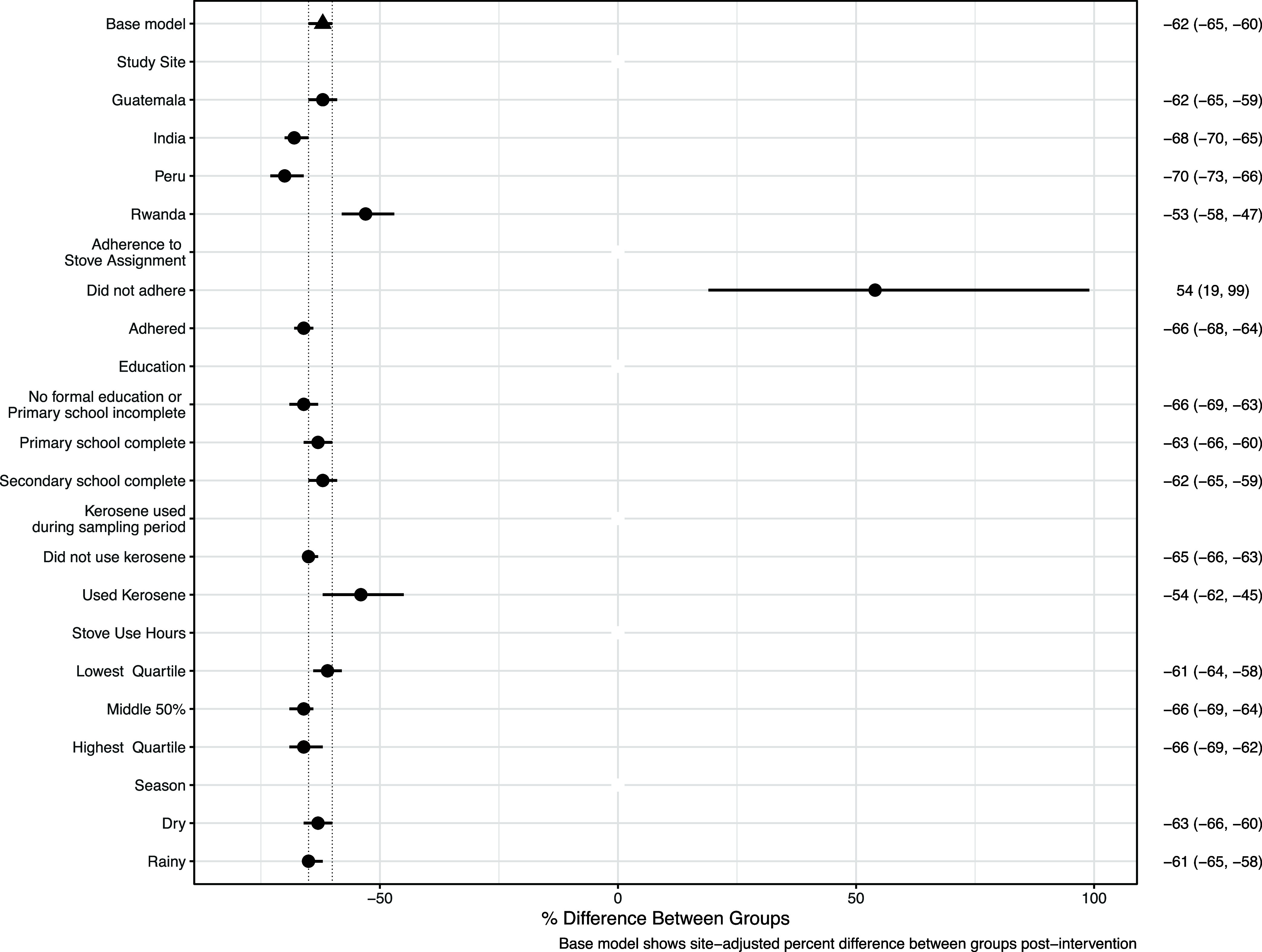
Hapin-wide postrandomization BC exposure contrasts (with 95% confidence intervals) between treatment arms (control vs intervention) by selected factors. Effect estimates outside of the confidence intervals reported in Johnson et al. 2022 (triangle) show how select factors potentially modified the effectiveness of the intervention in reducing personal exposures to BC. The percent differences in personal BC exposure between treatment arms were calculated within each subvariable using the emmeans package in *R*, which computes and compares marginal means.

HAPIN-wide, postintervention BC exposure contrasts between arms, by select factors, are shown in Figure 2; site-specific exposure contrasts are shown in Figures S6–S8. We found no potential for effect modification in Guatemala. Sample sizes for the control and intervention measures for each comparison are provided in Table S5. As reported by Johnson et al.^37^ (base model), BC exposures in the intervention group were significantly lower (−62, 95% CI: −65, −60) than those in the control group across all HAPIN sites. This contrast between groups was altered for participants who resided in Peru (−70, 95% CI: – 73, 66) and Rwanda (−53, 95% CI: −58, −47) and for participants who did not adhere to the assigned stove type (54, 95% CI: −19, 99) as confidence intervals for these effect estimates did not overlap that from Johnson et al.^37^ In India, the exposure contrast between groups was attenuated for kerosene users (−51, 95% CI: −62, −36) and enhanced when the humidity was in the lowest quartile (−77, 95% CI: −81, −72) compared to the BC exposure contrast reported by Johnson et al.^37^ (−69, 95% CI: −73, −66). In Peru, we estimated that the BC exposure contrast increased (from −62, 95% CI: −66, −57 to −72, 95% CI: −75, −69) after restricting to participants who adhered to the study stove assignment. We also found that the BC exposure contrasts between groups were enhanced for participants who used their stoves longer. In Rwanda, we observed the contrast between groups to be attenuated among participants who were in the “other” occupation category (i.e., participants did not work at home, as a vendor, or in agriculture) (−33, 95% CI: −49, −12).

## Discussion

### Study Overview

We conducted one of the largest and most comprehensive BC exposure assessment studies to date, comprising 7165 24 h measures from 3103 pregnant women across four diverse countries. We observed differences in personal exposures to BC according to primary stove type and kerosene use. In HAPIN-wide and site-specific analyses, we consistently observed a strong gradient where exposures were highest among participants using open fires, followed by those using improved biomass, than those using LPG stoves, respectively. Elevated exposures to BC were observed among kerosene users, compared to nonusers, for participants in Guatemala, India, and Rwanda, which highlights the potential importance of kerosene in these settings. These findings also support the notion that adherence to the HAPIN study stove assignment and general kerosene use can potentially impact the BC exposure contrast between treatment arms postrandomization; these effects were most prominent for participants in Peru and India, respectively.

Our study-wide and country-specific models performed moderately well (*R*^2^ range: 0.45–0.50) with *R*^2^ values similar to that reported for predicting personal PM_2.5_ exposures using only survey-based data in Kenya (*R*^2^ = 0.51)^48^ and higher than that reported for predicting global personal BC with survey data, ambient PM_2.5_, and household concentration levels (*R*^2^ range: 0.33–0.39),^32^ which highlights the explanatory power of information from questionnaires. Future studies may improve the predictive power of exposure models by more accurately characterizing the temporal changes in study participants’ activities in real-world conditions. Of particular importance is how certain temporal changes in time activity patterns are associated with combustion-related sources of air pollution like indoor smoking, garbage burning, crop burning, or the use of biomass for heating.

We also observed greater within-participant variance compared to between-participant variance in BC exposures, even after adjusting for key predictors of exposure. The ICC in our covariate-adjusted HAPIN-wide model (0.20) was higher than those observed for personal PM_2.5_ (0.16) and BC (0.11) exposures among adults in China,^35^ lower than that observed for carbon monoxide (CO) exposure among children in The Gambia (0.27),^49^ and overlapped with ICCs observed for exposures among adult women in Guatemala^50^ (CO: 0.17–0.33) and peri-urban India^51^ (PM_2.5_: 0.22; BC: 0.21). The direct comparison of ICCs across studies can be misleading given the differences in the predictors,^52^ target population, and pollutants assessed. Our ICCs, along with those from other longitudinal studies, suggest that a single 24 h exposure measurement does not sufficiently characterize longer-term exposures to investigate chronic health effects.

### Stove and Fuel as Predictors for Black Carbon Exposures

We observed substantial variation in personal BC according to stove and fuel types as shown in effect estimates from the overall (Figures 1 and S2) and site-specific (Figure S3) plots of multivariable linear regression coefficients. A previous assessment of household BC in eight LMICs observed a concentration gradient for household BC comparing gas to coal (+53% increase), wood (+142% increase), and other biomass (+190% increase) fuels.^32^ Although not completely comparable, we observed similar findings according to stove/fuel types where BC exposures increased going from LPG to other biomass stove users. The distinction between these stove types is driven primarily by the fuel type and combustion conditions associated with each stove. For example, in Rwanda, Imbabura stove users predominantly cooked with charcoal and had lower BC exposures than those burning wood with Rondereza or open-fire stoves. This finding is in line with both field-^39^ and lab-based studies,^53^ which suggest that charcoal has a lower BC emission factor (reported as quantities of emitted BC per unit of fuel used) than wood. We observed no significant differences in BC exposures among wood and cow dung users in Peru (Table S3), possibly suggesting that these two fuel types have similar burning efficiencies.

Our assessment of the stove type used during sampling allowed us to evaluate how the exposure contrast between arms changed after adjusting for adherence to the study stove assignment. HAPIN-wide, we found that adherence potentially modified the BC exposure contrast between groups; this effect was most pronounced in Peru. The percentage of Peruvian participants in control homes exclusively using an LPG stove in the past 24 h at baseline, postintervention visit 1, and postintervention visit 2 was 6, 22, and 23%, respectively. Although these percentages are much smaller than those for corresponding participants in intervention homes that reported exclusive LPG use at follow-up (96–97%),^54^ we estimated that the BC exposure contrast between arms would be greater by about 12% had participants adhered to stove assignments in Peru.

### Kerosene Use as a Predictor for Personal Black Carbon

Kerosene was an important driver of BC exposures in our data, likely due to the relatively high BC emission factor of this combustion source. Kerosene reportedly emits PM_2.5_ comprised mostly of BC (88–100%),^55^ which highlights the importance of studying the impact kerosene has on BC exposures in resource-limited areas of LMICs. The percentage of women with access to electricity for our study population in Rwanda (∼35%) was relatively higher than estimates in rural sub-Saharan Africa (SSA) (12%),^56^ yet much lower than that among women from the other HAPIN study sites (>90%).^48^ The lower access to electricity in Rwanda possibly suggests an increased reliance on fuel-based lighting sources like kerosene lamps and may explain why the magnitude of the association between kerosene use and BC exposure was greater than that seen in the other HAPIN IRCs. The elevated BC exposures seen among kerosene users in Rwanda were in line with those seen in Mozambique when comparing women using kerosene (+81%) to women using electricity as a lighting source.^34^ In Uganda, interventions of solar lighting systems versus fuel-based lighting sources resulted in massive reductions (>90%) in BC exposures,^57^ which suggests that exposures from fuel-based lighting sources can reduce the impact of a clean burning cookstove intervention on BC exposures. Future intervention programs may benefit from aiming to mitigate both cookstoves and lighting sources in tandem to comprehensively reduce exposures in resource-limited areas of LMICs, particularly in SSA.

The role of kerosene use for pregnant women in India is less clear than it is for those in Rwanda. For example, virtually all of our participants in India either had access to electricity or used electricity as a primary lighting source;^37^ however, ∼20% of participants used kerosene during sampling at baseline. While this percentage remained relatively constant in the control arm postrandomization, the percentage of those using kerosene nearly halved in the intervention arm postrandomization (Table S6). Additionally, unlike in Rwanda, both kerosene-related variables (e.g., general kerosene use during sampling and primary lighting source) were selected in the multivariable predictive model for India, which possibly alludes to multiple uses of kerosene in this study site. These assertions, along with technicians’ observations of Indian mothers using kerosene as a fire starter in the control group for cooking, imply that the intervention had an unforeseen benefit in reducing the need to use kerosene in the intervention arm postrandomization. Both kerosene-related variables that we assessed have the potential to modify the BC exposure contrast between treatment arms; however, we observed the general kerosene use variable to impact the intervention substantially in India where the exposure contrast was sharply attenuated among kerosene users.

### Study Limitations

The HAPIN study design included many strengths, such as robust sample size, global representation among LMICs, clear distinctions of stove and fuel use, repeated measures of personal BC, and comprehensive assessment of individual and group-level factors that can potentially impact exposures; our study also has some weaknesses. First, the current study only has two repeated postrandomization measures for individuals in the LPG stove intervention arm, limiting our ability to assess the within-participant variance among this group. Second, we have missing survey data; however, after conducting the same analysis with imputed data for missing variables, both nonimputed and imputed model results were similar to one another. Third, our questionnaires were not specific enough to discern the different uses of kerosene among our study population. However, we provided empirical evidence supporting our field exposure team’s suspicion that participants in India additionally used kerosene to ignite biomass or as a backup cooking fuel as opposed to using kerosene solely as a lighting source like participants in the other study sites. Future exposure assessments may benefit from administering more specific surveys to parse the potential impacts of exposure to kerosene from cooking and lighting practices. Fourth, combustion-related sources other than the cookstove (i.e., crop and waste burning, use of biomass for heating, indoor smoking, and other relevant sources) were identified in our questionnaire, yet we found no significant associations between these sources and BC likely due to the constraints of our HAPIN study design (e.g., eligibility criteria and selection of study sites with minimal ambient sources). Finally, HAPIN purposefully selected study sites that reduced the contribution of air pollution from traffic sources. Additionally, ambient measures of exposures were not available during the time of our data analysis for this work. Therefore, our results do not account for this source’s contribution to exposures among our study population and may impact the portability of our findings to other settings with significant traffic- or industry-related source profiles.

### Implications for Future Cookstove Interventions

The current study provides one of the most comprehensive assessments of personal BC exposures in LMICs. Our exposure modeling may provide important information to future studies aiming to mitigate and quantify the health risks associated with exposure to BC in settings where household air pollution is dominated by cookstove sources. This information can be used in future exposure and health studies to estimate exposures among this demographic on a grander scale than personal monitoring would allow. Furthermore, we identified several factors that can impact exposure to BC and explored how select factors potentially modified the effect of an LPG intervention in reducing exposure to BC. Our findings highlight the importance of both stove adherence as well as the mitigation of kerosene use in cookstove intervention trials to achieve more effective exposure contrasts between treatment groups. In conclusion, the information gleaned from the current study may be leveraged before and during future studies to implement more impactful cookstove intervention trials.

## Supporting Information Available

Description of individual and household characteristics (Text S1); description of imputation analysis (Text S2); summary statistics of personal BC (μg/m^3^) overall and by select factors (Table S1); postintervention personal BC (μg/m^3^) by treatment arm and IRC (Table S2); HAPIN-wide and IRC-specific association between personal BC and select factors (Table S3); exposure summary and model performance mixed-effects analysis with imputed data (Table S4); sample sizes (N measures) of comparison groups (control vs intervention) postintervention (Table S5); number (%) of baseline and postrandomization measures by treatment arm and kerosene use in India (Table S6); percent of missing data for each factor (Figure S1); Guatemala-specific multivariable linear regression coefficients (Figure S2); India-specific multivariable linear regression coefficients (Figure S3); Peru-specific multivariable linear regression coefficients (Figure S4); Rwanda-specific multivariable linear regression coefficients (Figure S5); postintervention BC exposure contrasts between treatment arms in India (Figure S6); postintervention BC exposure contrasts between treatment arms in Peru (Figure S7); postintervention BC exposure contrasts between treatment arms in Rwanda (Figure S8) (PDF)
