# Supplementary material for: Factors Determining Black Carbon Exposures among Pregnant Women Enrolled in the HAPIN Trial
Source: Environ Sci Technol. 2024 May 29;58(23):10162–74. doi: 10.1021/acs.est.3c09991 (PMC11171448; doi:10.1021/acs.est.3c09991)
Supplement: Supplementary file 1 — es3c09991_si_001.pdf [file es3c09991_si_001.pdf]

Supporting Information For

Factors Determining Black Carbon Exposures Among Pregnant Women Enrolled in the HAPIN Trial

Authors: Devan A. Campbell[1,15], Michael Johnson[2], Ricardo Piedrahita[2], Ajay Pillarisetti[3], Lance A. Waller[4], Katherine A. Kearns[1,2], Jacob Kremer[1], Erick Mollinedo[1], Jeremy A. Sarnat[4], Maggie L. Clark[5], Lindsay J. Underhill[6], John P. McCracken[1,7], Anaité Diaz-Artiga[7], Kyle Steenland[8], Ghislaine Rosa[9], Miles A. Kirby[10], Florien Ndagijimana[11], Ephrem Dusabimana[11], Kalpana Balakrishnan[12], Sankar Sambandam[12], Krishnendu Mukhopadhyay[12], Saritha Sendhil[12], Amudha Natarajan[12], Lisa M. Thompson[8,12], William Checkley[13,14], Laura Nicolaou[13,14], Stella Hartinger[13,14], Jennifer L. Peel[5], Thomas F. Clasen[8], Luke P. Naeher[1*]

Affiliations: [1]University of Georgia, Athens, GA, USA; [2]Berkeley Air Monitoring Group, Berkeley, CA, USA; [3]Environmental Health Sciences, School of Public Health, University of California, Berkeley, CA, USA; [4]Department of Biostatistics and Bioinformatics, Rollins School of Public Health, Emory University, Atlanta, GA, USA; [5]Department of Environmental and Radiological Health Sciences, Colorado State University, Fort Collins, CO.; [6]Washington University School of Medicine, St. Louis, MO, USA; [7]Center for Health Studies, Universidad del Valle de Guatemala, Guatemala City, Guatemala.; [8]Gangarosa Department of Environmental Health, Rollins School of Public Health, Emory University, Atlanta, GA, USA; [9]Department of Public Health, Policy and Systems, University of Liverpool, Liverpool, UK; [10]Department of Global Health and Population, Harvard T.H. Chan School of Public Health, Boston, MA USA; [11]ICMR Center for Advanced Research on Air quality, Climate and Health, Department of Environmental Health Engineering, Sri Ramachandra Institute of Higher Education and Research, Chennai, India.; [12]Nell Hodgson Woodruff School of Nursing, Emory University, Atlanta, GA, USA.; [13]Center for Global Non-Communicable Diseases, Johns Hopkins University, Baltimore, MD, USA; [14]Division of Pulmonary and Critical Care, School of Medicine, Johns Hopkins University, Baltimore, MD, USA.; [15]Benchmark Risk Group, Chicago, IL, USA

*Corresponding author: Luke Naeher; lnaeher@uga.edu, 150 East Green Street Rm 132, Athens, GA

27 Pages

Contains 2 texts, 6 tables, and 9 figures.

Text S1. Description of individual and household characteristics
Text S2. Description of imputation analysis
Table S1. Summary statistics of personal BC (μg/m^3) overall and by select factors
Table S2. Post-intervention personal BC (μg/m^3) by treatment arm and IRC
Table S3. HAPIN-wide and IRC-specific association between personal BC and select factors
Table S4. Exposure summary and model performance mixed effects analysis with imputed data
Table S5. Sample sizes (N measures) of comparison groups (Control vs Intervention) postintervention
Table S6. Number (%) of baseline and post-randomization measures by treatment arm and kerosene use in India
Figure S1. Percent of missing data for each factor

Figure S2. Guatemala-specific multivariable linear regression coefficients
Figure S3. India-specific multivariable linear regression coefficients
Figure S4. Peru-specific multivariable linear regression coefficients
Figure S5. Rwanda-specific multivariable linear regression coefficients
Figure S6. Postintervention BC exposure contrasts between treatment arms in India
Figure S7. Postintervention BC exposure contrasts between treatment arms in Peru
Figure S8. Postintervention BC exposure contrasts between treatment arms in Rwanda
References

Text S1. Description of individual and household characteristics

Primary stove types were categorized as open fires or LPG stoves, chimney stoves, or other improved biomass stoves such as portable biomass stoves, comals (i.e., smooth, flat griddles typically used in Central America), charcoal burning Imbabura stoves, and wood burning Rondereza stoves. Our questionnaires were not specific enough for us to discern whether kerosene fuel was used explicitly for lighting or cooking activities during the sampling period, although we had information on whether kerosene lamps were used as the primary lighting source. The self-reported other sources of smoke variable were categorized into none, neighbor's kitchen, and other, which includes smoke from either trash burning, tobacco smoke, agricultural burning, generators, mosquito coils, and other non-specific potential sources of smoke. Family size was categorized by number of individuals living in the home where small families had less than or equal to four individuals, medium-sized families had greater than four and less than ten individuals, and large families had greater than or equal to ten individuals living in the home. Food insecurity was obtained from the Food Insecurity Scale, developed by the Food and Agriculture Organization of the United Nations.[1] Roof and wall materials were dichotomized into impermeable (e.g., brick, cement, stone, wood, corrugated metal) and permeable (e.g., reed, thatch, mesh, wattle) materials. Kitchens were either located in the participant's bedroom or adjacent to the bedroom with or without a partition (inside), outside the participant's home with an enclosure (outside enclosed) or without an enclosure (outside open-air), or away from the participant's home (not at residence). Kitchen volume was calculated by taking the product of the kitchen length, width, and height. Temperature and relative humidity were obtained directly from the personal air monitor. In Guatemala, spring (March to May), summer (June to August) and fall (September to November) are representative of increased rainfall while winter (December to February) is distinguished by dry and mild conditions. In India, summer (March to May) is characterized intense heat and limited rainfall, spring (June to August) and fall (September to November) coincide with the monsoon periods, and winter (December to February) represents another dry season. In Peru, rainfall peaks in the summer (December to February), with moderate conditions in the fall (March to May), decreasing temperature and rainfall in the winter (June to August), and moderate temperature and drier conditions in the spring (September to November). In Rwanda, spring (March to May) is marked by increased humidity and rainfall, summer (July to August) comes with a drop in rainfall with a slight rise in temperature, fall (September to October) experiences a resurgence in rainfall, and winter (December to February) has steady rainfall with a relatively warm climate.

Text S2. Description of imputation analysis

As a sensitivity analysis, we imputed missing questionnaire data with the MICE package in R[2] and used a stepwise method similar to that posed in Brand[3] for imputed data to identify predictors of personal BC. Briefly, we imputed data for missing survey variables 10 times. Next, we performed stepwise elimination model selection for each imputed dataset separately, keeping all variables that were present in at least half of the 10 models. We then conducted a backward elimination procedure using the Wald statistic to test whether each variable should be in the final model. We removed each variable in turn and then compared models with and without the variable. If the Wald statistic had a p-value above 0.05, the variable was removed. This

backward elimination procedure stops when all p-values are less than 0.05. Model results using the imputed dataset are provided in Table S4.

**Table S1.** Summary statistics of personal BC (μg/m^3) overall and by select factors					
**Variable**	**N (measures)**	**(%)**	**Median (IQR)**	**Mean (SD)**	**Range**
**Overall**	7165	100%	7.1 (2.9 - 12.6)	9.3 (9.5)	0.6 - 132.6
**Primary stove**					
Chimney	357	5%	9.8 (5.6 - 13.6)	10.8 (8.3)	1.5 - 65.4
Imbabura	290	4%	6.9 (4.8 - 9.4)	7.8 (5.1)	2.7 - 43.2
LPG	2443	34%	2.7 (1.6 - 4.6)	4.0 (5.3)	0.6 - 131.5
Open fire	3515	49%	10.8 (6.4 - 15.5)	12.6 (10.6)	0.6 - 132.6
Other	240	3%	8.7 (4.5 - 13.8)	10.6 (9.7)	0.7 - 73.2
Rondereza	320	4%	11.1 (7.8 - 14.5)	12.3 (8)	2.8 - 76.9
**Participant cooked**					
No	474	7%	4.3 (2.3 - 8.4)	7 (8.6)	0.7 - 97.8
Yes	6672	93%	7.3 (2.9 - 12.8)	9.5 (9.6)	0.6 - 132.6
Missing	19	0%	4.2 (2.1 - 10.3)	6.1 (4.8)	1.1 - 14.6
**Participant used kerosene fuel**					
No	6704	94%	6.9 (2.8 - 12.2)	8.8 (8.6)	0.6 - 132.6
Yes	432	6%	11.3 (5.5 - 20.8)	16.5 (17.2)	0.7 - 122
Missing	29	0%	3.7 (1.8 - 9)	5.6 (4.7)	1.1 - 14.6
**Other sources of smoke reported by the participant**					
None	6598	92%	6.9 (2.8 - 12.5)	9.2 (9.6)	0.6 - 132.6
Neighbor kitchen	427	6%	9.4 (5.2 - 13.7)	10.9 (9.4)	0.7 - 85.7

Other	35	0%	3.8 (1.7 - 11.6)	6.3 (5.5)	1.1 - 23.3
Missing	105	1%	8.7 (4.2 - 11.6)	10.3 (9.2)	0.8 - 66.1

Table S2. Post-intervention personal BC (μg/m^3) by treatment arm and IRC

	N (measures)	%	Median (IQR)	Mean (SD)	Range
**Control**					
HAPIN	2266	100	9.6 (5.2 - 14)	11.0 (9.9)	0.7 - 120
Guatemala	640	28	11.0 (8.2 - 15)	12.0 (6.9)	2.5 - 88
India	581	26	8.7 (4.4 - 14)	11.0 (11.0)	0.7 - 99
Peru	447	20	4.1 (1.6 - 12)	8.6 (12.0)	1.3 - 120
Rwanda	598	26	10 (6.8 - 14)	12.0 (9.1)	2.8 - 120
**Intervention**					
HAPIN	2360	100	2.8 (1.6 - 4.8)	4.1 (5.5)	0.6 - 130
Guatemala	685	29	2.8 (2.6 - 5.5)	4.9 (6.4)	2.2 - 130
India	594	25	2.3 (1.3 - 3.9)	3.9 (6.3)	0.6 - 110
Peru	510	22	1.6 (1.5 - 1.6)	2.0 (1.5)	1.4 - 14
Rwanda	571	24	4.1 (2.9 - 6)	5.4 (5.0)	2.5 - 55

**Table S3.** HAPIN-wide and IRC-specific association between personal BC and select factors										
	**HAPIN[a]**		**Guatemala[b]**		**India[b]**		**Peru[b]**		**Rwanda[b]**	
	% Change (95%CI) in Personal BC	**R^2** or Sample Size	% Change (95%CI) in Personal BC	**R^2** or Sample Size	% Change (95%CI) in Personal BC	**R^2** or Sample Size	% Change (95%CI) in Personal BC	**R^2** or Sample Size	% Change (95%CI) in Personal BC	**R^2** or Sample Size
**IRC**		**0.08**		---		---		---		---
Guatemala	Ref	2000	---	---	---	---	---	---	---	---
India	-26 (-31, -21)	1874	---	---	---	---	---	---	---	---
Peru	-48 (-52, -45)	1553	---	---	---	---	---	---	---	---
Rwanda	-3 (-9, 4)	1738	---	---	---	---	---	---	---	---
**Cooking fuel[c]**		**0.06**		---		---		**0**		**0.21**
Wood	Ref	1848	Ref	---	Ref	---	Ref	73	Ref	407
Charcoal	---	152	---	---	---	---	---	---	-47 (-52, -41)	152
Cow Dung	---	514	---	---	---	---	1 (-21, 30)	514	---	---
Other fuel[d]	-31 (-38, -23)	19	---	---	---	---	-2 (-53, 105)	8	-17 (-42, 20)	9
**Primary stove used during sampling**		**0.42**		**0.48**		**0.32**		**0.4**		**0.36**
Open fire	Ref	3515	Ref	1016	Ref	1229	Ref	808	Ref	462
Chimney	-19 (-26, -12)	357	-23 (-28, -16)	294	-37 (-75, 62)	3	2 (-17, 26)	60	---	0
Imbabura[f]	---	---	---	---	---	---	---	---	-41 (-46, -36)	290
Rondereza[f]	---	---	---	---	---	---	---	---	-10 (-17, -3)	320
Other stove[e]	-35 (-39, -30)	850	-36 (-46, -22)	29	-21 (-36, -3)	64	-51 (-63, -35)	31	-19 (-27, -10)	116
LPG	-70 (-71, -69)	2443	-67 (-68, -65)	661	-73 (-75, -71)	578	-75 (-77, -73)	654	-62 (-65, -59)	550
**Participant cooked**		**0.08**		**0**		**0**		**0**		**0.02**
No	Ref	474	Ref	39	Ref	90	Ref	202	Ref	143

Yes	20 (11, 30)	6672	-8 (-26, 14)	1954	22 (-3, 52)	1782	10 (-6, 30)	1346	41 (26, 58)	1590
**Kitchen location**		**0.1**		**0**		**0.01**		**0.02**		**0.23**
Inside	Ref	4192	Ref	1641	Ref	1477	Ref	473	Ref	601
Outside enclosed	29 (23, 36)	2209	9 (-1, 20)	317	26 (11, 43)	345	1 (-10, 14)	848	96 (84, 110)	699
Outside open-air	66 (52, 80)	638	43 (2, 99)	12	-1 (-36, 51)	22	57 (31, 86)	197	101 (86, 118)	402
Kitchen not at residence	-34 (-62, 15)	9	106 (-46, 685)	1	---	---	-59 (-82, -6)	6	41 (-36, 213)	2
**Primary lighting source**		**0.08**		**0**		**0.01**		**0**		**0.09**
Electricity	Ref	5503	Ref	1770	Ref	1802	Ref	1424	Ref	507
Kerosene lamp	92 (65, 125)	183	15 (-24, 75)	16	107 (40, 203)	38	114 (-73, 1595)	1	121 (92, 156)	128
Other	10 (-1, 22)	409	2 (-10, 16)	175	---	---	-6 (-30, 26)	59	38 (21, 57)	175
Solar light	15 (4, 27)	616	-9 (-59, 97)	4	-30 (-70, 65)	8	1 (-27, 40)	49	30 (19, 42)	555
Torch (battery)	6 (-5, 19)	436	-12 (-37, 23)	23	-11 (-45, 43)	26	-33 (-60, 11)	19	23 (12, 36)	368
**Kerosene used during sampling**		**0.1**		**0**		**0.05**		**0**		**0.05**
No	Ref	6704	Ref	1959	Ref	1546	Ref	1536	Ref	1663
Yes	82 (67, 99)	432	12 (-12, 43)	33	85 (63, 110)	322	83 (-16, 301)	7	110 (79, 146)	70
**Other sources of smoke**		**0.08**		**0**		**0**		**0**		**0.01**
None	Ref	6598	Ref	1720	Ref	1846	Ref	1498	Ref	1534
Neighbor kitchen	11 (2, 21)	427	4 (-5, 14)	252	10 (-36, 88)	14	34 (-32, 166)	9	22 (9, 35)	152
Other	-24 (-43, 1)	35	-31 (-55, 5)	10	-54 (-79, 4)	6	-11 (-49, 58)	13	2 (-39, 72)	6
**Participant Occupation**		**0.1**		**0.01**		**0.06**		**0**		**0.07**
Agriculture	Ref	3247	Ref	13	Ref	762	Ref	1187	Ref	1285
Commercial	-32 (-39, -23)	282	7 (-36, 77)	43	-25 (-63, 51)	11	8 (-21, 49)	51	-37 (-44, -29)	177

Household	-31(-36, -26)	3251	44 (-8, 125)	1859	-41 (-47, -35)	1042	-7 (-21, 8)	228	-31 (-40, -20)	122
Other	-20 (-28, -10)	363	63 (0, 164)	69	-20 (-41, 8)	59	2 (-20, 31)	86	-30 (-39, -20)	149
**Family size**		**0.08**		**0**		**0**		**0**		**0**
Small (<=4)	Ref	4622	Ref	977	Ref	1360	Ref	879	Ref	1406
Medium (5-9)	1 (-4, 6)	2333	-1 (-8, 7)	857	9 (-4, 23)	508	1 (-10, 13)	652	-6 (-15, 4)	316
Large (>10)	1 (-13, 19)	191	-9 (-21, 5)	154	6 (-59, 177)	6	71 (4, 183)	20	31 (-19, 112)	11
**Access to electricity**		**0.08**		**0**		**0**		**0**		**0.05**
No	Ref	1456	Ref	205	Ref	72	Ref	84	Ref	1095
Yes	-19 (-24, -12)	5632	-7 (-18, 4)	1783	-29 (-47, -5)	1802	11 (-13, 42)	1468	-27 (-32, -21)	579
**Household food insecurity**		**0.08**		**0**		**0**		**0**		**0.02**
None	Ref	4075	Ref	1115	Ref	1515	Ref	794	Ref	651
Mild	11 (5, 17)	1899	3 (-5, 13)	613	19 (1, 39)	266	7 (-6, 21)	540	23 (12, 35)	480
Moderate/Severe	12 (4, 20)	1073	12 (-1, 26)	234	14 (-13, 49)	84	5 (-12, 25)	200	17 (7, 28)	55
**Age at baseline**		**0.08**		**0**		**0**		**0.01**		**0**
<20	Ref	896	Ref	299	Ref	300	Ref	187	Ref	110
20-24	-9 (-16, -2)	2717	0 (-10, 12)	804	-8 (-22, 7)	902	-21 (-35, -6)	560	-15 (-28, 1)	451
25-29	-4 (-11, 4)	2243	8 (-4, 22)	580	-14 (-28, 2)	526	-4 (-21, 15)	504	-8 (-22, 8)	633
30-35	-6 (-14, 3)	1291	5 (-8, 21)	305	-21 (-37, 1)	146	-9 (-26, 12)	301	-8 (-22, 8)	539
**Participant education**		**0.09**		**0.01**		**0.02**		**0.01**		**0.06**
No complete formal education or Primary school incomplete	Ref	2387	Ref	943	Ref	656	Ref	62	Ref	726
Primary school complete	-17 (-21, -11)	2490	-7 (-14, 1)	789	-26 (-35, -15)	540	-36 (-53, -15)	463	-11 (-17, -3)	698

Secondary school or equivalent completed	-23 (-28, -18)	2285	-15 (-24, -5)	266	-28 (-37, -18)	678	-29 (-47, -6)	1027	-38 (-43, -31)	314
**Roof material**		**0.07**		**0**		**0.02**		**0**		**0.01**
Impermeable	Ref	5006	Ref	1894	Ref	981	Ref	888	Ref	1243
Permeable	-13 (-18, -7)	1332	-6 (-21, 13)	86	-26 (-34, -17)	877	8 (-6, 25)	348	49 (8, 105)	21
**Wall material**		**0.07**		**0**		**0**		**0**		**0**
Impermeable	Ref	5173	Ref	1723	Ref	1135	Ref	1095	Ref	1220
Permeable	-2 (-9, 5)	1103	4 (-7, 17)	240	-8 (-18, 2)	693	12 (-9, 36)	138	-5 (-27, 22)	32
**Season**		**0.08**		**0**		**0**		**0**		**0.05**
Dry	Ref	1907	Ref	475	Ref	304	Ref	792	Ref	336
Rainy	-11 (-16, -7)	5258	0 (-7, 7)	1525	-6 (-16, 7)	1570	-10 (-19, 0)	761	-31 (-36, -26)	1402
**Hours of stove use per day**	7 (5, 8)	**0.09**	3 (2, 5)	**0.01**	26 (21, 31)	**0.07**	5 (1, 8)	**0**	6 (4, 8)	**0.02**
		7041		1976		1843		1518		1704
**Relative humidity (per 5%)**	-7 (-9, -7)	**0.1**	-7 (-9, -5)	**0.02**	-8 (-10, -6)	**0.03**	-6 (-8, -4)	**0.02**	-8 (-10, -7)	**0.04**
		6829		1858		1859		1418		1694
**Temperature (per 5 degrees Celsius)**	7 (3, 12)	**0.08**	24 (15, 34)	**0.02**	-10 (-18, -2)	**0**	7 (-2, 17)	**0**	29 (19, 42)	**0.02**
		6829		1858		1859		1418		1694
**Kitchen volume (per 10m^3)**	0 (0, 0)	**0.07**	-2 (-3, -1)	**0.01**	-3 (-6, 0)	**0**	0 (0, 0)	**0**	-5 (-9, -1)	**0.01**
		7048		1976		1844		1524		1704

[a] HAPIN-wide models are adjusted for IRC

[b] IRC-specific univariable analysis

[c] Cooking fuel analysis only includes baseline measures

[d] For HAPIN-wide analysis only, other fuel includes charcoal and cow dung

[e] For HAPIN-wide analysis only, other stove includes Imbabura and Rondereza stoves, as well as stoves reported as “other”

[f] Imbabura and Rondereza stoves included in Rwanda model analysis only

[g] Marginal R^2 (**bold**) represents the percentage of variation explained by fixed effects

[h] Sample size shows the number of observations per category with a valid personal BC measurement

Table S4. Exposure summary and model performance mixed effects analysis with imputed data

Study Site	Model parameters	Sample Size	Median ($\mu g/m^3$)	Mean ($\mu g/m^3$)	SD ($\mu g/m^3$)	RMSE ($\mu g/m^3$)	ICC	Marginal R^2
HAPIN	Study site + primary stove type + secondary stove type + participant cooked + other sources of smoke + primary lighting source + general kerosene use + stove use hours + kitchen location + occupation roof material + wall material + education + humidity + season	7165	6.9	7.6	4.6	6.8	0.21	0.47
Guatemala	Primary stove type + secondary stove type + other sources of smoke + kitchen volume + education + humidity + season	2000	9.7	8.9	4.1	6.5	0.25	0.50
India	Primary stove type + participant cooked + primary lighting source + general kerosene use + stove use hours + occupation + wall material + humidity temperature + season	1874	6.4	7.3	4.8	7.0	0.18	0.44
Peru	Primary stove type + secondary stove type + participant cooked + stove use hours + kitchen location + family size + age at baseline	1553	4.5	5.2	3.7	7.8	0.23	0.45

Rwanda	Primary stove type + participant cooked + other sources of smoke + primary lighting fuel + general kerosene use + stove use hours + occupation + education + humidity + season	1738	8.2	8.6	4.3	6.0	0.14	0.47

SD: Standard deviation; ICC: Intraclass Correlation; RMSE: Root Mean Square Error

**Table S5.** Sample sizes (N measures) of comparison groups (Control vs Intervention) postintervention									
		HAPIN		India		Peru		Rwanda	
Predictors		N (Control measures)	N (Intervention measures)	N (Control measures)	N (Intervention measures)	N (Control measures)	N (Intervention measures)	N (Control measures)	N (Intervention measures)
	Johnson et al. 2022	2266	2360	581	594	447	510	598	571
Study site									
	Guatemala	640	685	---	---	---	---	---	---
	India	581	594	---	---	---	---	---	---
	Peru	447	510	---	---	---	---	---	---
	Rwanda	598	571	---	---	---	---	---	---
Adherence									
	No	129	90	9	26	119	18	---	---
	Yes	2137	2270	572	568	328	498	---	---
Participant cooked									
	No	142	153	---	---	---	---	---	---
	Yes	2118	2197	---	---	---	---	---	---
Hours of stove use during sampling									
	Lowest Quartile	823	1023	---	---	190	153	302	372
	Middle 50%	611	524	---	---	201	276	225	172
	Highest Quartile	832	813	---	---	56	81	71	27
Roof material									
	Impermeable	1523	1795	---	---	---	---	---	---

	Permeable	429	378	---	---	---	---	---	---
General kerosene use									
	No	2104	2261	459	539	---	---	---	---
	Yes	152	86	118	53	---	---	---	---
Kitchen location									
	Inside	1117	1801	---	---	---	---	---	---
	Outside enclosed	796	496	---	---	---	---	---	---
	Outside open-air	314	9	---	---	---	---	---	---
	Kitchen not at residence	3	5	---	---	---	---	---	---
Participant Occupation									
	Agriculture	---	---	233	253	---	---	478	394
	Commercial	---	---	5	2	---	---	45	68
	Household	---	---	324	321	---	---	28	54
	Other	---	---	19	18	---	---	45	53
Other sources of smoke reported by the participant									
	None	---	---	---	---	---	---	534	520
	Neighbor kitchen	---	---	---	---	---	---	44	35
	Other	---	---	---	---	---	---	2	1
Food insecurity									
	None	---	---	---	---	---	---	188	242
	Mild	---	---	---	---	---	---	177	155
	Moderate/Severe	---	---	---	---	---	---	216	157

Season									
	Dry	606	653	109	113	---	---	95	99
	Rainy	1660	1707	472	481	---	---	503	472

Table S6. Number (%) of baseline and post-randomization measures by treatment arm and kerosene use in India

Kerosene use	Control		Intervention	
	Baseline	Post-randomization	Baseline	Post-randomization
No	275 (78)	459 (80)	273 (78)	539 (91)
Yes	76 (22)	118 (20)	75 (22)	53 (9)
Total	351 (100)	577 (100)	348 (100)	592 (100)

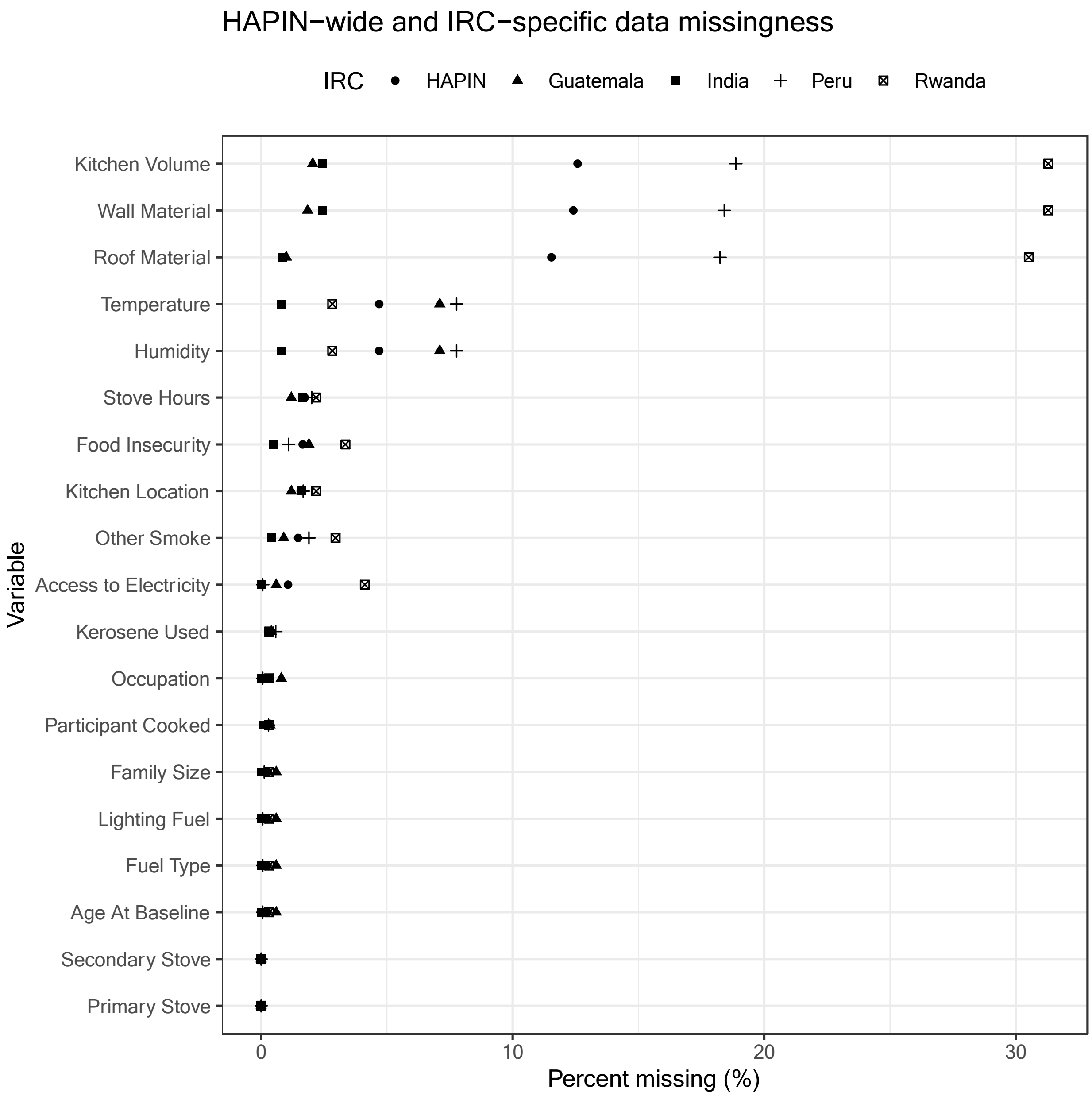

Figure S1. Percent of missing data for each covariate in HAPIN (circle), Guatemala (square), Peru (plus), and Rwanda (box with a check).

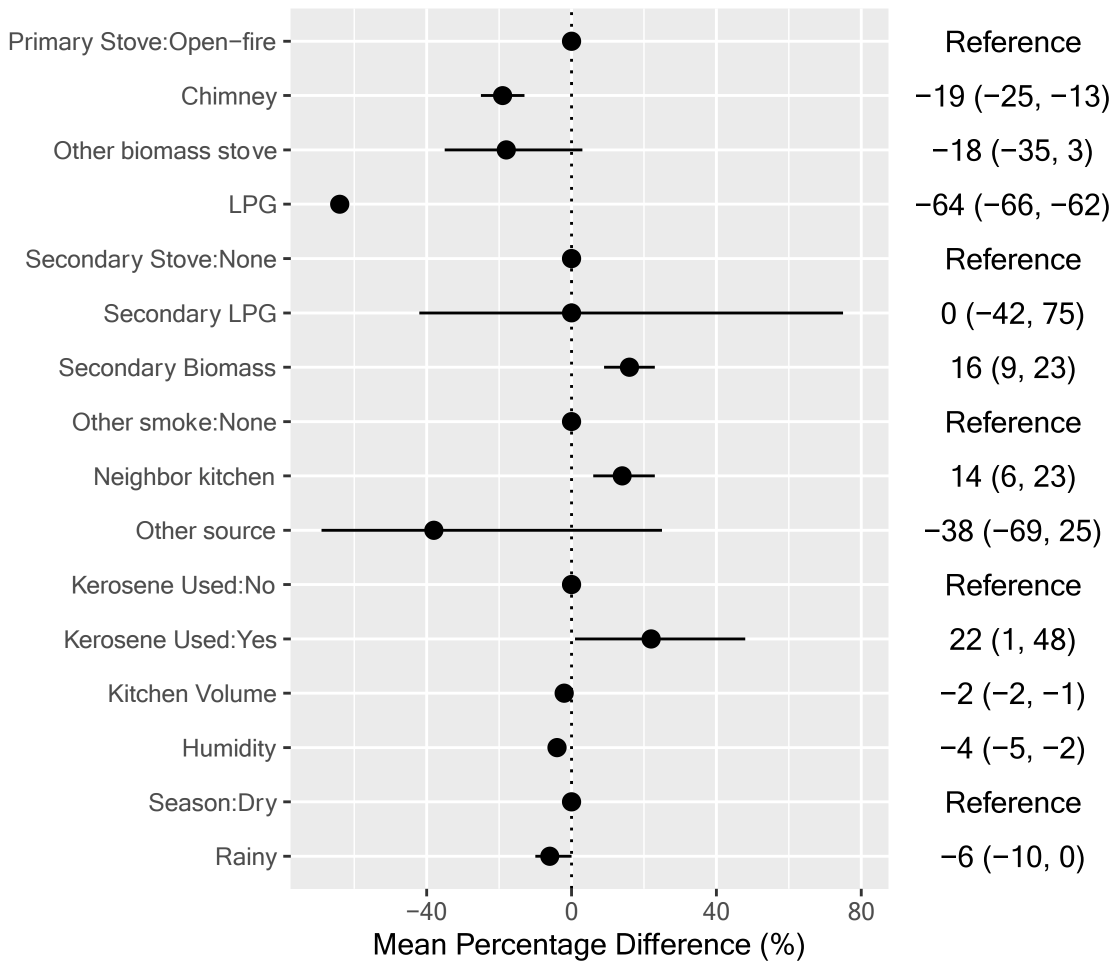

Figure S2. Guatemala-specific multivariable linear regression coefficients (with 95% confidence intervals). Numeric coefficients represent the mean percentage change of the geometric mean on respective BC exposures compared to the reference category based on the final multivariable linear regression models. Coefficients for relative humidity and kitchen volume represent a 5 unit increase in percentage and 10 unit increase in volume, respectively.

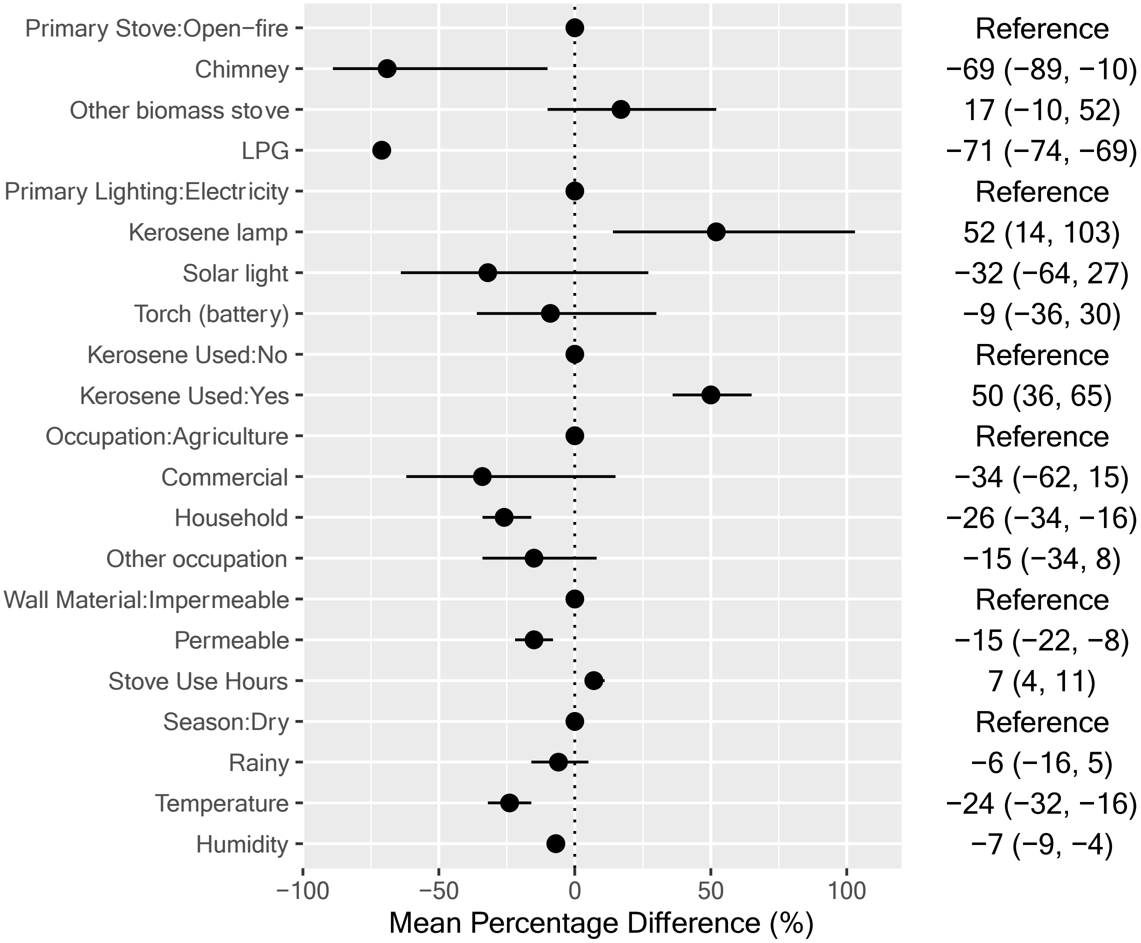

Figure S3. India-specific multivariable linear regression coefficients (with 95% confidence intervals). Numeric coefficients represent the mean percentage change of the geometric mean on respective BC exposures compared to the reference category based on the final multivariable linear regression models. Coefficients for relative humidity and temperature represent a 5 unit increase in percentage and degrees Celsius, respectively.

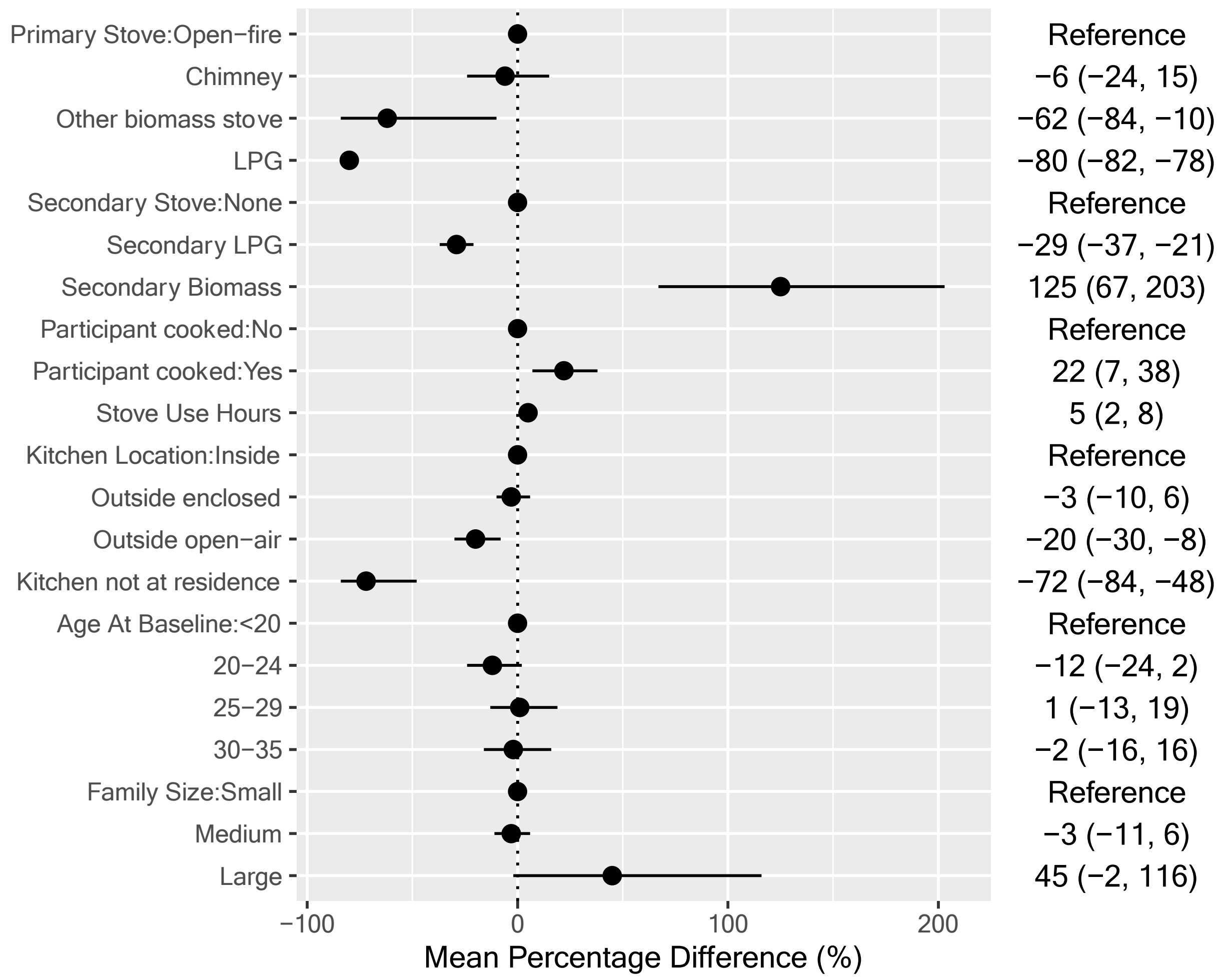

Figure S4. Peru-specific multivariable linear regression coefficients (with 95% confidence intervals). Numeric coefficients represent the mean percentage change of the geometric mean on respective BC exposures compared to the reference category based on the final multivariable linear regression models. Coefficients for relative humidity, temperature, and kitchen volume represent a 5 unit increase in percentage, a 5 unit increase in degrees Celsius, and a 10 unit increase in volume, respectively.

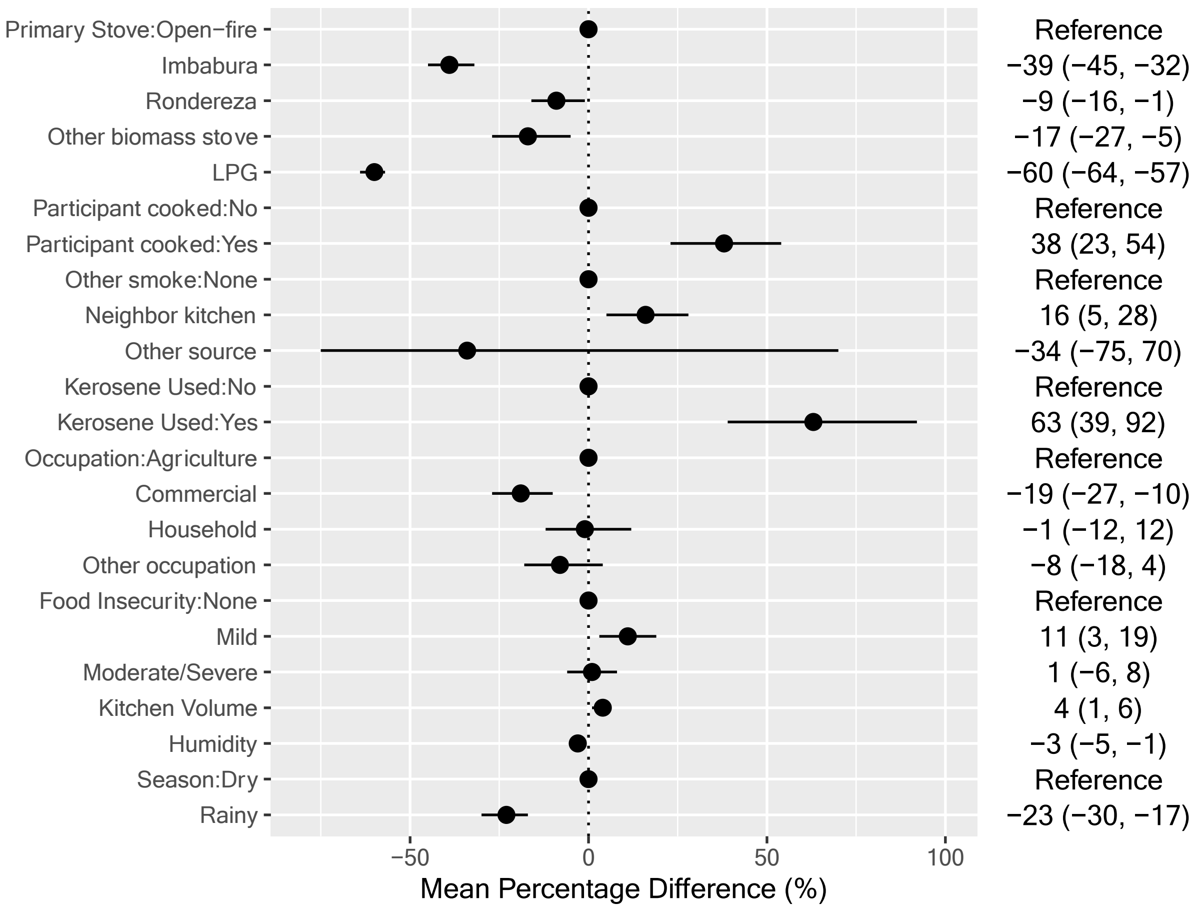

Figure S5. Rwanda-specific multivariable linear regression coefficients (with 95% confidence intervals). Numeric coefficients represent the mean percentage change of the geometric mean on respective BC exposures compared to the reference category based on the final multivariable linear regression models. Coefficients for relative humidity and kitchen volume represent a 5 unit increase in percentage and 10 unit increase in volume, respectively.

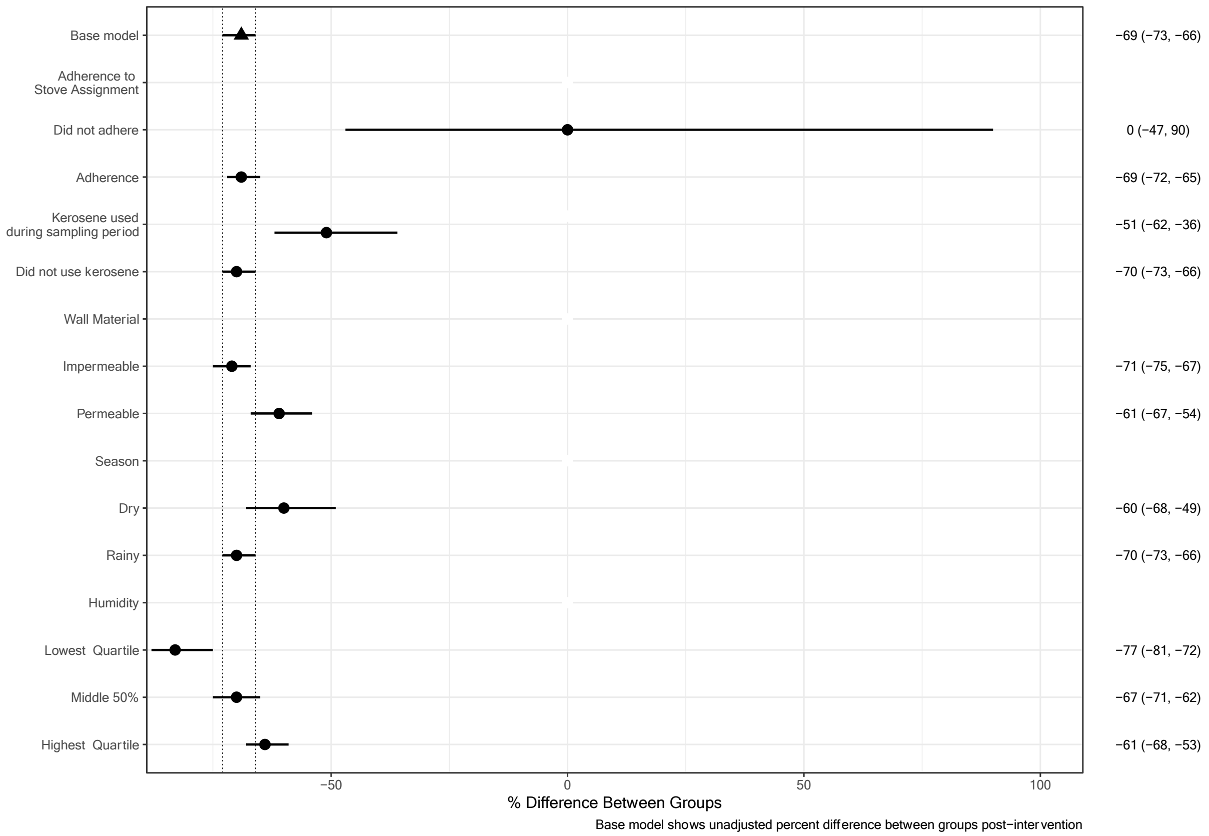

Figure S6. Postintervention BC exposure contrasts (with 95% confidence intervals) between treatment arms (Control v Intervention) by selected factors in India. Effect estimates outside of the confidence intervals reported in Johnson et al. 2022 (triangle) show how select factors potentially modified the effectiveness of the intervention in reducing personal exposures to BC. The percent differences in personal BC exposure between treatment arms were calculated within each sub-variable using the emmeans package in R which computes and compares marginal means.

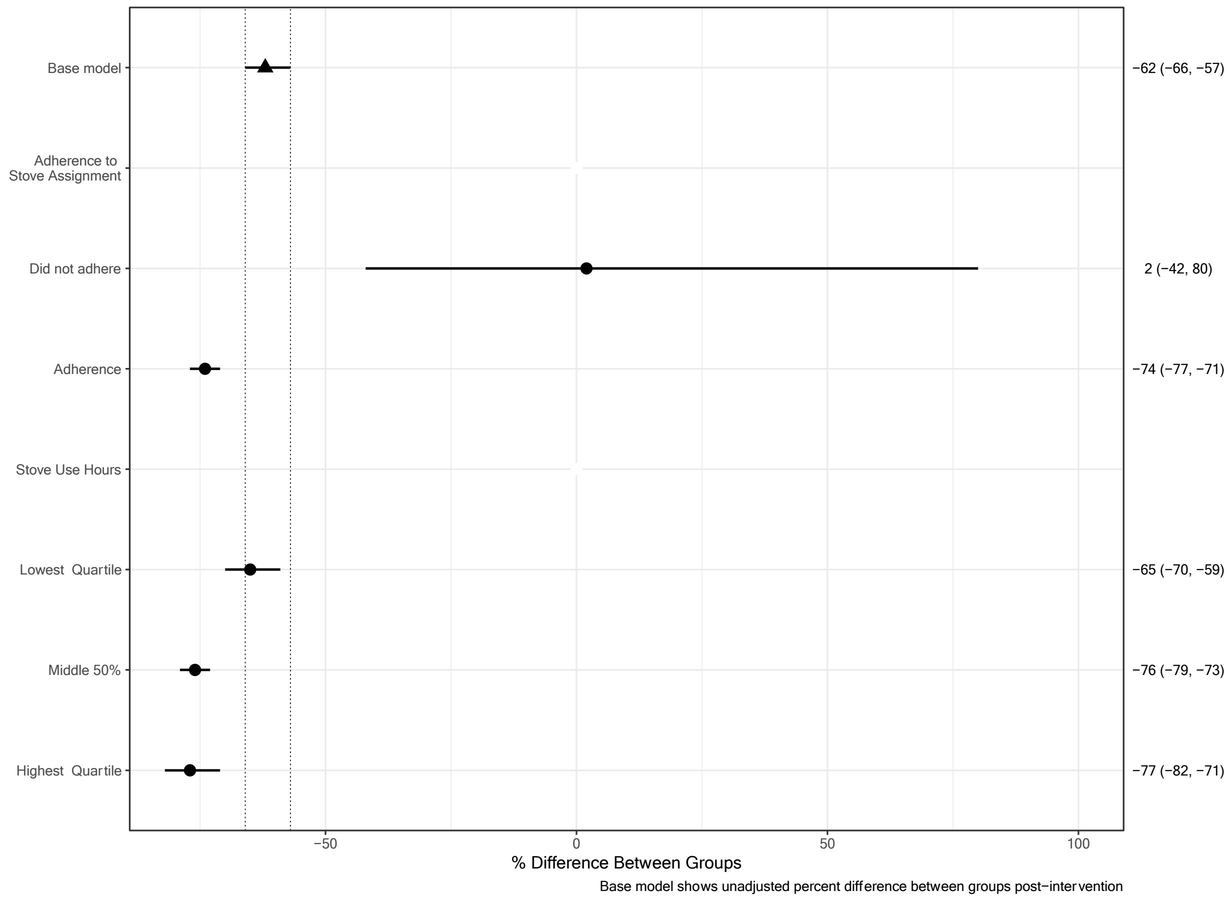

Figure S7. Post-randomization BC exposure **contrasts** (with 95% confidence intervals) between treatment arms (Control v Intervention) by selected factors in Peru. Effect estimates outside of the confidence intervals reported in Johnson et al. 2022 (triangle) show how select factors potentially modified the effectiveness of the intervention in reducing personal exposures to BC. The percent differences in personal BC exposure between treatment arms were calculated within each sub-variable using the emmeans package in R which computes and compares marginal means.

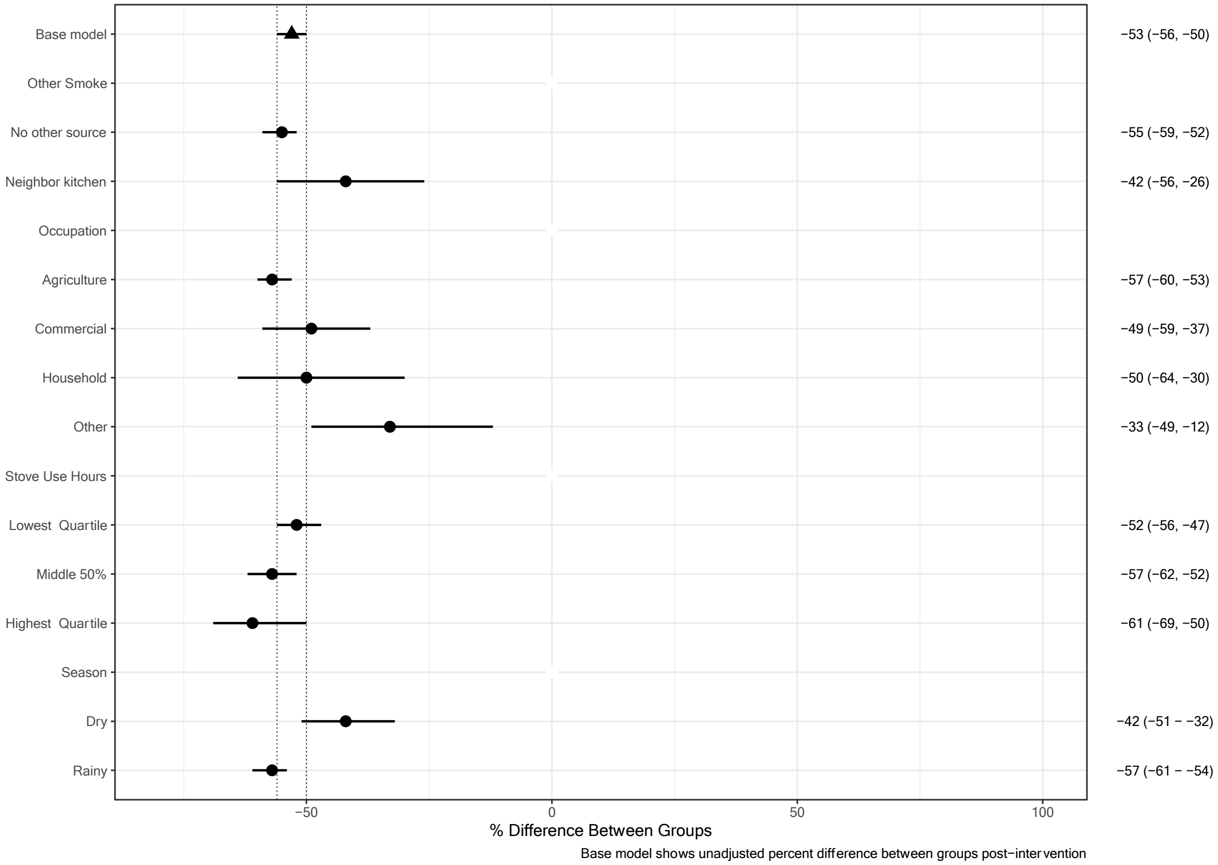

Figure S8. Post-randomization BC exposure contrasts (with 95% confidence intervals) between treatment arms (Control v Intervention) by selected factors in Rwanda. Effect estimates outside of the confidence intervals reported in Johnson et al. 2022 (triangle) show how select factors potentially modified the effectiveness of the intervention in reducing personal exposures to BC. The percent differences in personal BC exposure between treatment arms were calculated within each sub-variable using the emmeans package in R which computes and compares marginal means.

References

1. Ha, S. *et al.* Ambient air pollution and the risk of pregnancy loss: a prospective cohort study. *Fertil. Steril.* **109**, 148–153 (2018).
2. Buuren, S. van & Groothuis-Oudshoorn, K. mice: Multivariate Imputation by Chained Equations in R. *J. Stat. Softw.* **45**, 1–67 (2011).
3. Brand, J. *Development, Implementation and Evaluation of Multiple Imputation Strategies for the Statistical Analysis of Incomplete Data Sets*. (1999).
